# Physiological and Morphological Responses of Blackberry Seedlings to Different Nitrogen Forms

**DOI:** 10.3390/plants12071480

**Published:** 2023-03-28

**Authors:** Yongkang Duan, Haiyan Yang, Hao Yang, Zhiwen Wei, Jilu Che, Wenlong Wu, Lianfei Lyu, Weilin Li

**Affiliations:** 1 Co-Innovation Center for Sustainable Forestry in Southern China, College of Forestry, Nanjing Forestry University, Nanjing 210037, China; dyk@njfu.edu.cn (Y.D.); yanghao_19940720@163.com (H.Y.); wzw0709wzw@163.com (Z.W.); jlche@njfu.edu.cn (J.C.); 2 Institute of Botany, Jiangsu Province and Chinese Academy of Sciences (Nanjing Botanical Garden Mem. Sun Yat-sen), Jiangsu Key Laboratory for the Research and Utilization of Plant Resources, Nanjing 210014, China; 1964wwl@163.com (W.W.); njbglq@163.com (L.L.)

**Keywords:** blackberry, nitrogen, ammonium, nitrate, plant growth, physiological parameters

## Abstract

Blackberries are an emerging third-generation fruit that are popular in Europe, and specific nitrogen (N) supply is an important factor affecting their growth and development. To study the optimal N fertilizer for blackberry seedlings, no N (CK), nitrate (NO_3_^−^)–N, ammonium (NH_4_^+^)–N and urea were applied to one-year-old ‘Ningzhi 4’ blackberry plants at a key growth period (from May to August) to explore the effects of different N forms on the physiological characteristics. Correlation and principal component analysis were used to determine the relationships between various indexes. Ammonium (NH_4_^+^) or urea-fed plants had a better growth state, showed a greater plant height, biomass, SPAD values and enhanced antioxidant enzyme activities and photosynthesis. In addition, NH_4_^+^ was beneficial to the accumulation of sugars and amino acids in leaves and roots, and promoted the transport of auxin and cytokinin to leaves. NO_3_^−^ significantly inhibited root growth and increased the contents of active oxygen, malondialdehyde and antioxidants in roots. Correlation and principal component analysis showed that growth and dry matter accumulation were closely related to the antioxidant system, photosynthetic characteristics, amino acids and hormone content. Our study provides a new idea for N regulation mechanism of blackberry and proposes a scientific fertilization strategy.

## 1. Introduction

The blackberry plant (*Rubus* spp.), native to North America, is a perennial shrub of the genus *Rubus* in the Rosaceae [1]. The berries are aggregated fruits that are black when ripe and look like mulberries. Blackberry fruit is rich in anthocyanins, superoxide dismutase (SOD), flavonoids, ellagic acid, tannins and other antioxidants, and has the highest concentration of selenium, which can prevent the occurrence of a range of diseases among a range of tested fruits [2]. In addition, blackberry leaves have been reported to have antibacterial, antiseptic and antidiarrheal properties in traditional medicine, as well as the ability to facilitate labor during pregnancy [3]. With the increasing demand for high-quality and nutritious fruits that improve health, blackberry acreage is increasing. The global commercial cultivated area for blackberry was estimated to increase by 34.92% from 20,035 ha in 2005 to 27,032 ha in 2015 [4]. The global annual output of commercial blackberry exceeds 150,000 tons per year [2].

Nitrogen (N) is an essential element and plays a crucial role in the yield and quality of blackberry fruit. In production practice, farmers usually use N fertilizers in large quantities to increase the productivity of crops. However, unreasonable N fertilizer application will not only make crops over-reliant on chemical fertilizers but also lead to destruction of the soil structure and soil nutrient imbalance [5]. In addition, excess N fertilizer accelerates the pollution of the atmosphere and water resources through surface runoff, leaching and ammonia (NH_3_) volatilization [6,7]. Therefore, in large-scale cultivation of blackberries, the prudent application of N fertilizer, improvement of N fertilizer use efficiency and maximization of economic output are of great significance for achieving sustainable agricultural development.

Plants obtain a variety of N forms from the soil that undergo a series of processes, such as transport, assimilation, storage and remobilization [8]. Ammonium (NH_4_^+^) and nitrate (NO_3_^−^) are the main forms of N taken up by plants [9], with specific transporters required for their uptake [10]. Urea is often used in agricultural production due to its stable chemical properties and because it has the highest N content among organic N fertilizers. The majority of urea applied as fertilizer is degraded to NH_4_^+^ by urease derived from soil bacteria and transported into the plant. Urea that escapes urease degradation is transported into the plant by a high-affinity transport system [11].

There are great differences in N form preference among different plants. The N form preference depends on not only the species and developmental stage, but also a variety of environmental factors, such as temperature, moisture and light [12,13]. In general, NO_3_^−^ may be more beneficial during a drought, as NH_4_^+^ is the primary source of N take up in flooded, freeze-damaged and acidic soils [10]. Taking into account assimilation costs, NH_4_^+^ is the preferred N source for most plants, due to its low metabolic cost for assimilation [9]. However, most plants are sensitive to NH_4_^+^, and long-term application of NH_4_^+^ usually inhibits plant growth and may be toxic [10]. The toxicity of NH_4_^+^ to plants depends on its concentration, and the concentration at which toxicity becomes apparent varies among plant species. Only a few plant species can tolerate high concentrations of NH_4_^+^, such as tea tree, sugarcane, rice and blueberry [6,14,15,16]. A deficiency of NO_3_^−^ may also be another NH_4_^+^ stress, when NH_4_^+^ is fed as a single N source, in addition to the toxicity issue of NH_4_^+^ itself. An increasing number of studies have shown that the negative effects of NH_4_^+^ on plants can be alleviated by feeding NO_3_^−^ [17,18,19]. The Columbia strain of *Arabidopsis thaliana* cannot grow normally when fed with NH_4_^+^ as a single N source. However, by adding NO_3_^−^ or pre-culturing in a medium containing NO_3_^−^, *Arabidopsis thaliana* can grow well and absorb NH_4_^+^ rapidly [18]. In addition, plants fed with urea as the sole N source may also have the same toxic symptoms as NH_4_^+^. This effect may be related to the NH_4_^+^ produced by urea hydrolysis [19]. In the study of wheat seedlings, it was found that NO_3_^−^ could also promote the assimilation rate of urea and NH_4_^+^, which had a positive effect on plant cells to alleviate NH_4_^+^ poisoning [17]. It should be noted that even plants that are obviously tolerant to NH_4_^+^ will have symptoms of poisoning as long as the concentration of NH_4_^+^ exceeds the threshold they can bear. For example, under the condition of excessive NH_4_^+^ feeding, the leaves of rice will gradually turn yellow, the plants will be thin and growth will be inhibited. Only when NO_3_^−^ and NH_4_^+^ are supplied together can the growth of rice be restored [19]. Another reason for NH_4_^+^ toxicity may also be that root acidification hinders cell metabolism. Generally speaking, NH_4_^+^-tolerant plants are more adaptable to acidic environment [8,19]. Although it may have adverse effects on most plants under high NH_4_^+^ conditions, the existence of NH_4_^+^ as the main N source may have important positive significance for plants. A large number of studies have reported that NH_4_^+^ nutrition can stimulate N assimilation, promote photosynthetic electron transport and regulate specific secondary metabolic pathways [6,14,19]. In addition, some plants fed with NH_4_^+^ also have a higher tolerance to abiotic stresses such as drought and salinity [12,13].

Different N forms have different effects on physiological and biochemical processes such as photosynthesis, antioxidant system regulation and N metabolism [20]. Cruz et al. [21] showed that NO_3_^−^ promoted photosynthesis during later stages of cassava growth. Some scholars believe that NH_4_^+^ is beneficial for crop photosynthesis, but an excessively high concentration can easily lead to NH_4_^+^ toxicity in plants, and NO_3_^−^ can reduce the chlorophyll content of leaves and inhibit photosynthesis [16,22]. Hessini et al. [13] conducted a pot experiment on maize and found that NH_4_^+^ was more conducive to the accumulation of leaf dry matter than NO_3_^−^ and significantly improved stomatal conductance and the transpiration rate, as well as increased the amino acid and sugar contents in leaves. Studies have shown that NH_4_^+^ can alter the redox state of reactive oxygen species (ROS) and regulate the homeostasis of antioxidant enzyme systems [16]. After Arabidopsis plants were supplied with NH_4_^+^, the leaves exhibited symptoms of stress, and ROS levels increased [23]. Additionally, NH_4_^+^ can increase the activity of antioxidant enzymes in maize roots [24]. Notably, N forms also affect the levels of free amino acids and endogenous hormones. Researchers reported that when NH_4_^+^ was used as the sole N source, the total amino acid content in coffee seedlings was higher than that with NO_3_^−^-N treatment [25]. Similar results were found in rice and tea tree [26,27]. It is generally believed that NO_3_^−^ promotes the synthesis of cytokinin (CTK) in plants and increases the activity and transport rate of CTK, while NH_4_^+^ inhibits the transport of CTK from roots to aerial parts and reduces its content [28,29]. A study on maize roots showed that the inhibition of root growth by high NO_3_^−^ levels was caused by a reduction in the root auxin (IAA) content [30]. The increase in root biomass promoted by NH_4_^+^ and urea was related to the increase in the IAA content [31]. The study of N forms affecting the hormone content has only been conducted in a few plant species, and how N forms regulate the movement and distribution of IAA and CTK is still unclear.

To the best of our knowledge, the N form preference of blackberry has not been reported, and there has been no systematic in-depth study on the mechanism of the N adaptation strategy at the whole-plant level. As an emerging fruit, people mainly focus on the preservation, antioxidant and anthocyanin research of blackberry fruit. Moreover, blackberry plants are highly adaptable and have relatively low nutrient requirements compared to other fruits [32], so there is little research on cultivation of blackberry. Based on the above, understanding the preference for N fertilizers is necessary for blackberry fertilization programs.

The experiment was carried out using potted plants after the addition of different N fertilizers. In this study, we performed an in-depth analysis of the N absorption mechanism of blackberry plants at morphological and physiological levels. The purpose of our study was to investigate how the changes in morphological and physiological parameters of blackberry plants depend on different N forms. Another goal was to try to evaluate the availability of different N forms on the growth and development of blackberry seedlings and determine the best fertilization strategy. This study provides some new clues for the study of the mechanism of plant N absorption and utilization in the future.

## 2. Results

### 2.1. Growth Characteristics

**Figure 1:**
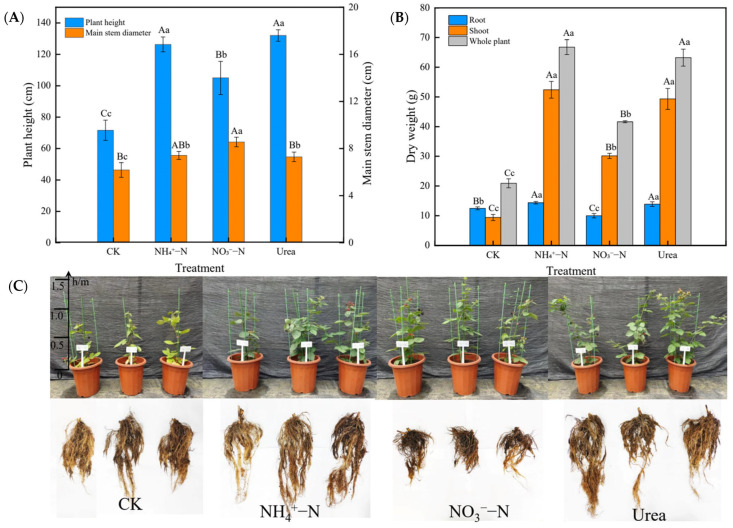
(**A**) Plant height and main stem diameter in response to different N forms. (**B**) The dry weights of the roots, shoots and whole plants in response to different N forms. (**C**) Phenotypes of the above- and below-ground parts of blackberry after 60 days of treatment with different N forms. Different letters show significant differences between treatments (uppercase letters indicate *p* < 0.01, lowercase letters indicate *p* < 0.05).

Different N forms significantly affected the aboveground and underground parts of the blackberry plants (Figure 1 showed a comparison of the NH_4_^+^–N, NO_3_^−^–N and urea treatments). Compared with other treatments, root growth was significantly inhibited with the NO_3_^−^–N treatment for 60 days (Figure 1B,C). The NH_4_^+^–N and urea treatments increased by 76.27% and 84.18%, respectively, in plant height (*p* < 0.01) relative to the controls (Figure 1A). The main stem diameter with the NH_4_^+^-N and urea treatments was similar to that under CK, but the NO_3_^−^–N treatment resulted in a significantly greater main stem diameter than the other treatments (*p* < 0.05). The NH_4_^+^–N (mean; 66.79 g DW/plant) and urea (mean; 63.24 g DW/plant) treatments increased the whole plant biomass more than the NO_3_^−^–N treatment (mean; 41.64 g DW/plant) (*p* < 0.01). This trend was consistent across the roots and shoots (Figure 1B).

### 2.2. Morphological Changes

**Figure 2:**
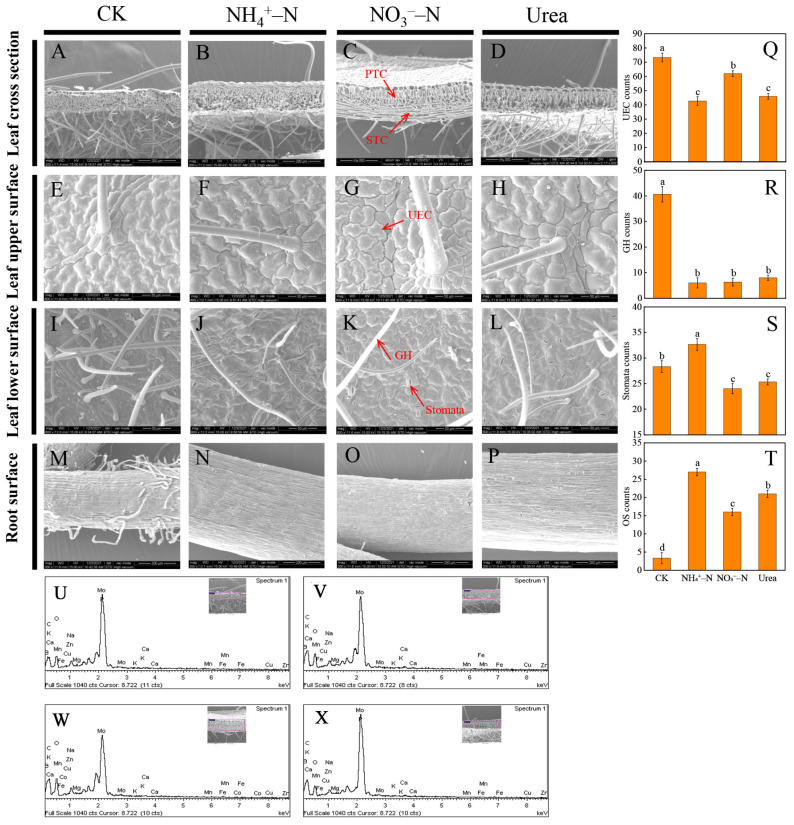
Scanning electron micrographs showing the leaf cross-sections ((**A**–**D**); scale bar is 200 μm), leaf upper surface ((**E**–**H**); scale bar is 50 μm), leaf lower surface ((**I**–**L**); scale bar is 50 μm) and root surface ((**M**–**P**); scale bar is 200 μm) after 60 days of treatment with different N forms. The counts of UEC (**Q**), GH (**R**), stomata (**S**) and OS (**T**) with different N forms. X-ray energy spectrogram of leaf cross-sections with different N treatments (panels (**U**–**X**) represent the CK, NH_4_^+^–N, NO_3_^−^–N and urea treatments, respectively). PTC, palisade tissue cell; STC, spongy tissue cell; UEC, upper epidermal cell; GH, glandular hairs; OS, open stomata.

In this study, the application of different N fertilizers significantly influenced the microstructure of blackberry leaves and roots (Figure 2). By observing the cross-section of the leaves, we found that the palisade tissue was more compact and denser in the NH_4_^+^–N and urea treatments, while the leaf was thicker with the NO_3_^−^–N treatment but the spongy tissue was looser compared to that under CK (Figure 2A–D). Upper epidermal cell (UEC) counts were significantly affected in the order CK > NO_3_^−^–N > urea > NH_4_^+^–N, indicating that within the same field of vision, the UEC area was larger with the NH_4_^+^–N and urea treatments (*p* < 0.05) (Figure 2E–H,Q). Compared with that under the other treatments, the lower surface of the leaves in the CK group had the most glandular hairs (GHs), but the single-stomata area was small and most stomata were closed. Among the three N treatments, NH_4_^+^–N resulted in the highest number of stomata and greater stomatal opening (Figure 2I–L,S,T). There were a high number of root hairs on the root surface in the CK group, and there was no significant difference among the other treatments (Figure 2M–P).

As shown in Figure 2U–X, the different N forms had no significant effect on the energy spectrum peaks of the leaf cross-section, but all the spectra showed that the peak for Mo was the highest (Kα = 4.5 keV). In each treatment, the leaves all contained C, O, Na and Ca, and Mg, K, Mn, Fe, Cu, Zn and Mo were also detected in some treatments (Table 1). The Na levels in leaves were the lowest with the NH4+–N treatment. The levels of Mg, Ca and Mn in NH_4_^+^–fed plants were significantly higher than those in other treatments. Interestingly, among the three N conditions, Fe, Cu and Mo were not detected in the NH_4_^+^–N treatment. The differences in the levels of other elements are shown in Table 1.

### 2.3. Cultivation Substrate Analysis

Compared with other treatments, the NO_3_^−^–N treatment significantly increased the cultivation substrate pH, and the NH_4_^+^–N treatment exhibited the lowest pH (*p* < 0.05). Different N forms had different effects on the electrical conductivity (EC) of the cultivation substrates, and the EC values were in the following order: NH_4_^+^–N > urea > NO_3_^−^–N (*p* < 0.05). The EC with the NH_4_^+^–N treatment was 1.89 times higher than that with the NO_3_^−^–N treatment. With the urea treatment, the substrate organic matter (SOM) and organic carbon (SOC) levels peaked, with mean values of 80.72% and 46.82%, respectively (Table 2). The content of alkali-hydrolysable nitrogen (AHN) in the cultivation medium was in the order NH_4_^+^–N > urea > NO_3_^−^–N > CK. Compared with that under the CK treatment, the AHN content increased by 41.99%, 29.93% and 19.72% with the NH_4_^+^–N, urea and NO_3_^−^–N treatments, respectively (*p* < 0.05).

### 2.4. Root Physiology and Antioxidant Indicators

**Figure 3:**
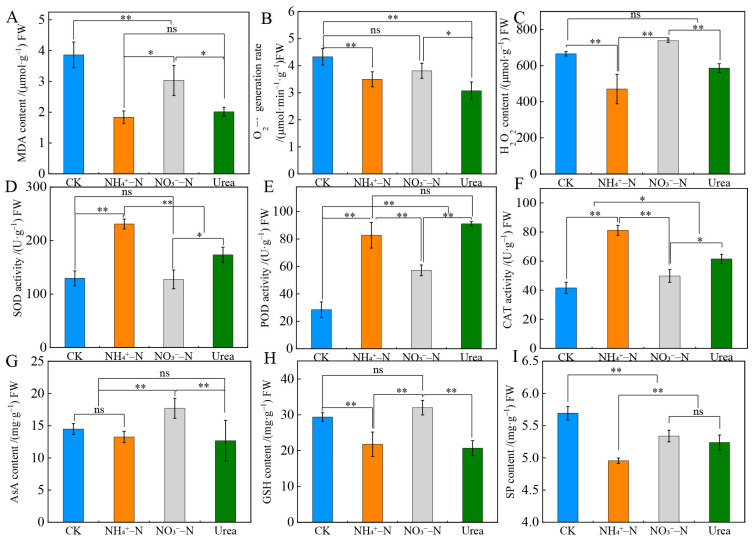
Changes in root physiology and antioxidant indicators with different N forms. The MDA (**A**), H_2_O_2_ (**C**), AsA (**G**), GSH (**H**) and SP (**I**) levels. O_2_^·−^ generation rate of (**B**). Activities of SOD (**D**), POD (**E**) and CAT (**F**). MDA, malondialdehyde; O_2_^·−^, superoxide anion radical; H_2_O_2_, hydrogen peroxide; SOD, superoxide dismutase; POD, peroxidase; CAT, catalase; AsA, ascorbic acid; GSH, reduced glutathione; SP, soluble protein. The data indicated are the means ± SDs (*n* = 3). Different letters show significant differences between treatments (** *p* < 0.01, * *p* < 0.05).

In the case of long-term zero N fertilizer (CK) treatment, the MDA and H_2_O_2_ levels and the O_2_^·−^ generation rate were high in the roots (Figure 3A–C). Notably, the antioxidant enzyme activity (including that of SOD, POD and CAT) was at the lowest level, but the levels of antioxidant substances (AsA and GSH) and the SP content remained relatively high under CK (Figure 3D–I). The O_2_^−^ generation rate and the levels of MDA and H_2_O_2_ with NO_3_^−^-N treatment were significantly higher than those with the NH_4_^+^–N and urea treatments, while the SOD, POD and CAT activities reached the lowest level [mean; 127.46 (U·g^−1^) FW, 57.14 (U·g^−1^) FW, 49.76 (U·g^−1^) FW]. Except for the SP content, which was not obviously different between the NO_3_^−^–N and urea treatments, the levels of AsA and GSH after NO_3_^−^–N treatment were significantly higher than those after NH_4_^+^–N and urea supply (*p* < 0.01). In summary, the above analysis showed that the roots were damaged by more free radicals with the CK and NO_3_^−^–N treatments.

### 2.5. Photosynthesis and Gas Exchange

**Figure 4:**
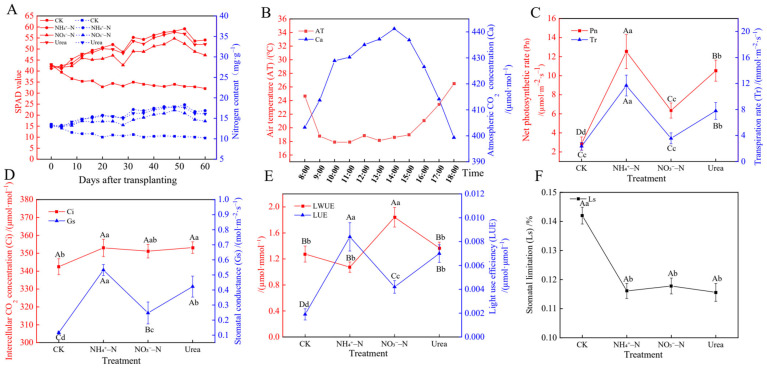
Effects of different N forms on the leaf photosynthetic parameters of blackberry. Changes in the relative chlorophyll content (SPAD value) and N content with time (**A**). Changes in air temperature (AT) and atmospheric CO_2_ concentration (Ca) during the day (**B**). Effects of different N forms on the net photosynthetic rate (Pn) and transpiration rate (Tr) (**C**), intercellular CO_2_ concentration (Ci) and stomatal conductance (Gs) (**D**), leaf water use efficiency (LWUE) and light use efficiency (LUE) (**E**) and stomatal limitation (Ls) (**F**). The data indicated are the means ± SDs (*n* = 20). Different letters show significant differences between treatments (uppercase letters indicate *p* < 0.01, lowercase letters indicate *p* < 0.05).

Figure 4A showed that with increasing treatment time, the relative chlorophyll content (SPAD value) and N content first increased and then decreased, peaking at approximately 52 days. Without N fertilization (CK), these levels gradually decreased. With the supply of NH_4_^+^–N, the SPAD value and N content increased, whereas the lowest values were measured in response to NO_3_^−^–N. Through real-time monitoring of changes in environmental factors, we found that the temperature peaked at 34 °C at 14:00 and was lowest at 18 °C at 18:00. At the same time, the atmospheric CO_2_ concentration (Ca) was stable at approximately 400 µmol·mol^−1^ during the day (Figure 4B). NH_4_^+^–N may positively affect the photosynthetic rate and gas exchange performance of blackberry plants (Figure 4C,D). Plants exhibited the highest net photosynthetic rate (Pn), transpiration rate (Tr) and stomatal conductance (Gs) with the NH_4_^+^–N treatment (*p* < 0.01), which were five times, three times and five times higher, respectively, than those under the CK treatment. With the NH_4_^+^–N treatment, the light use efficiency (LUE) was highest (mean; 0.0084 g µmol·µmol^−1^), while the leaf water use efficiency (LWUE) (mean; 1.84 g µmol·mmol^−1^) was the highest with the NO_3_^−^–N treatment (*p* < 0.01) (Figure 4E). The intercellular CO_2_ concentration (Ci) and stomatal limitation (Ls) did not change significantly among the N forms (Figure 4D,F).

### 2.6. Sugar and Endogenous Hormone Levels in Roots and Leaves

**Figure 5:**
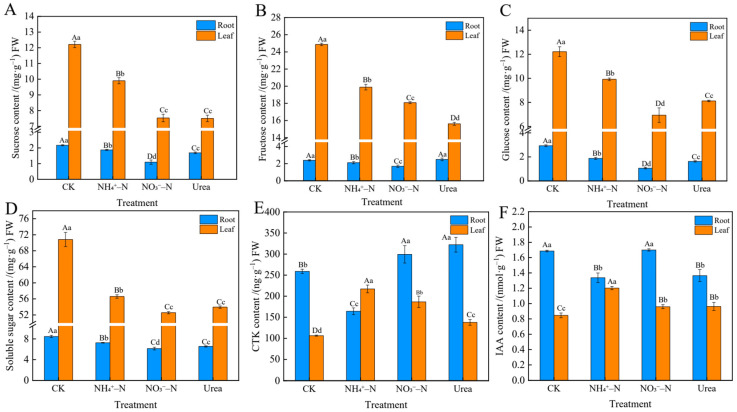
Effects of different N forms on sugar (**A**–**D**) and endogenous hormone contents (**E**,**F**) in roots and leaves. The data presented are the means ± SDs (*n* = 3). Different letters show significant differences between treatments (uppercase letters indicate *p* < 0.01, lowercase letters indicate *p* < 0.05).

Differences in N forms resulted in obvious differences in the accumulation and distribution of sugar and endogenous hormones (Figure 5). NH_4_^+^–N was more conducive to the accumulation of sugar in leaves and roots. The sucrose content in plants fed the NH_4_^+^–N nutrient solution was 31.47% and 32.00% higher in the leaves and 70.00% and 10.65% higher in the roots than in the respective NO_3_^−^–N and urea treatments (*p* < 0.01). Fructose was mainly distributed in leaves, exhibiting five times higher levels than in roots (Figure 5A). The change patterns of the fructose, glucose and soluble sugar levels showed a similar result (Figure 5B–D). Except for the fructose content, which was the lowest in leaves with the urea treatment, all sugars in leaves and roots showed the lowest levels with the NO_3_^−^–N treatment. In addition, CK plants accumulated high levels of various sugars, which may be related to N deficiency stress in plants. The CTK content in the leaves (Figure 5E) peaked at 217.18 ± 7.47 mg∙g^−1^ with the NH_4_^+^–N treatment (*p* < 0.01), while the CTK content in roots was highest with the urea treatment. Similarly, the NH_4_^+^–N treatment increased IAA content in leaves, while NO_3_^−^–N treatment led to the increase of IAA content in roots (Figure 5F).

### 2.7. Free Amino Acid Contents in Roots and Leaves

The content and distribution of free amino acids were also affected by different N forms. A total of 17 free amino acids were identified by an automatic amino acid analyzer, i.e., eight essential amino acids (EAAs) and nine nonessential amino acids (NEAAs) (Table 3). Compared with those with the CK treatment, the levels of the free amino acids with three N fertilizer treatments were higher, among which the free amino acid content was highest in roots with the NH_4_^+^–N treatment, followed by the NO_3_^−^–N treatment. In leaves, the content of the free amino acids was also the highest with the supply of NH_4_^+^–N, followed by urea treatment. In general, the content of free amino acids in leaves was much higher than that in roots with the different N treatments. In roots and leaves, the total amino acid (TAA) and NEAA levels were ranked in the following order: NH_4_^+^–N > urea > NO_3_^−^–N (*p* < 0.05). EAAs showed this order only in leaves. The predominant amino acid in roots supplied with NH_4_^+^–N was arginine, followed by serine, histidine and alanine, whereas with the NO_3_^−^–N supply, serine predominated, followed by glutamic acid, alanine and histidine, and with urea supply, arginine predominated, followed by alanine, histidine and phenylalanine. With the NH_4_^+^–N and urea treatments in leaves, the accumulation of serine, alanine and proline increased significantly and was higher than that with the NO_3_^−^–N treatment (*p* < 0.05), although the phenylalanine content was highest with the NO_3_^−^–N treatment.

### 2.8. Correlation and PCA of Physiological Indexes with Different N Forms

**Figure 6:**
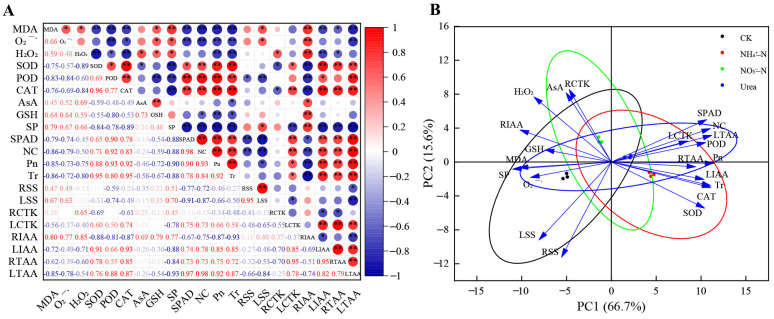
(**A**) Correlation matrix of the physiological indicators. The results were derived from the Pearson correlation analysis. NC, nitrogen content; RSS, soluble sugar in roots; LSS, soluble sugar in leaves; RCTK, cytokinin in roots; LCTK, cytokinin in leaves; RIAA, auxin in roots; LIAA, auxin in leaves; RTAA, total amino acid in roots; LTAA, total amino acid in leaves. * represents a significant correlation at the 0.05 level and ** represents a significant correlation at the 0.01 level. (**B**) PCA score chart of the physicochemical properties of blackberry with different N forms.

According to the correlation matrix analysis results (Figure 6A), the levels of MDA and H_2_O_2_ were significantly negatively correlated with the SOD, POD and CAT activities (*p* < 0.05). The SOD, POD and CAT activities were positively correlated with SP, SPAD, NC, Pn, LIAA and LTAA (*p* < 0.05). The SPAD, NC, Pn and Tr values were significantly positively correlated with LIAA, RTAA and LTAA (*p* < 0.01) and negatively correlated with the MDA content and O_2_^·−^ generation rate (*p* < 0.01). The other physiological indicators showed varying degrees of correlation.

Principal component analysis (PCA) showed two principal components, PC1 and PC2, and their eigenvalues were greater than 1. Table 4 showed that 66.72% and 15.64% of the variance was explained by PC1 and PC2, respectively. The cumulative variance contribution rate was 82.36% (>75%), so the two principal components accurately covered the information of 21 physiological indicators, with good data interpretation and high reliability. In addition, the scatter points corresponding to each treatment were obviously separated, indicating that different N forms had a significant impact on various indicators of blackberry (Figure 6B). The separation degree between the CK treatment and NH_4_^+^–N treatment was the largest, and the area of the intersection between the two circles (black and red ellipses) was the smallest, indicating that the difference between the CK and NH_4_^+^–N treatments was the most significant. The eigenvectors reflect the degree and direction of the influence of each trait on the principal component loading. On PC1, antioxidant enzyme activities, photosynthetic indicators, hormones (in leaves) and the total amino acid content had large positive coefficient values, indicating that these indicators had a large positive effect, whereas membrane lipid peroxide, GSH, SP and RIAA were negatively correlated. PC2 was significantly negatively correlated with RSS and LSS, whereas AsA and RCTK showed a significantly positive correlation (eigenvalue > 0.6, Table 4 and Figure 6B).

### 2.9. Correlation between Growth Indicators and Principal Components

It should be noted that the principal components PC1 and PC2 were independent and uncorrelated with each other. The relationship between the growth status of blackberry with different N forms and the physiological response could be analyzed by exploring the correlation between blackberry growth indexes and the two principal components. Data analysis indicated that all growth indicators showed a good positive correlation with principal component PC1, among which the significance of the plant height, shoot and whole plant dry weight was *p* < 0.01 and that of the main stem diameter and root dry weight was *p* < 0.05 (Table 5). In addition, except for root dry weight, which was significantly positively correlated with principal component PC2, the other growth indicators had no correlation. Therefore, principal component PC1 explained the growth of blackberry with different N treatments well. In other words, MDA, O_2_^·−^, H_2_O_2_, SOD, POD, CAT, GSH, SP, SPAD, NC, Pn, Tr, LCTK, RIAA, LIAA, RTAA and LTAA (marked with ** on PC1) were closely related to the blackberry response to different N forms.

## 3. Discussion

Our study was designed to evaluate the vegetative growth response of blackberry plants supplied with different N forms, which has not been well addressed in current large-scale commercial blackberry plantations. NH_4_^+^–N and NO_3_^−^–N are the most important forms of inorganic N absorbed by plants and have important effects on plant growth. The mainstream view is that urea, as an organic N form, must be catalyzed to NH_4_^+^ before it can be absorbed by plants [11]. In other words, NH_4_^+^ and urea share a common metabolic pathway, so it is critical to explore the preferences for the two inorganic N forms. In general, plants can adapt well when NO_3_^−^–N is supplied as the sole N source, even at high concentrations [33]. In contrast, when NH_4_^+^–N is supplied at relatively low concentrations, plants exhibit metabolic disorders such as membrane structure damage and disrupted osmoregulation systems [34]. An increasing number of studies have shown that this phenomenon is not absolute but is mainly related to plant species [35,36]. For example, it has been found that corn, tea tree, blueberry, etc., have better tolerance to NH_4_^+^–N [8,27,37]. Our research showed that blackberry grew better with the NH_4_^+^–N treatment, especially the roots (Figure 1C). However, NO_3_^−^–N treatment significantly inhibited root biomass accumulation, indicating that NH_4_^+^–N was more strongly associated with root growth. The assimilation cost of NH_4_^+^–N is reported to be lower than that of NO_3_^−^–N [38], which may be the primary reason many plants prefer NH_4_^+^–N to NO_3_^−^–N. Efficient uptake of NH_4_^+^–N may contribute to the long-term adaptation of plants to an NH_4_^+^–N environment, as our study showed that antioxidant enzyme (SOD, POD and CAT) activities were enhanced to maintain ROS and MDA at normal levels (Figure 3A–F). In addition, the increased photosynthetic rate (Figure 4C) made it possible for roots and leaves to accumulate more carbohydrates (glucose, fructose, etc.) (Figure 5A–D), which, together with the increased free amino acid content (Table 3), may improve the adaptation of blackberry to NH_4_^+^–N [10]. This is consistent with previous findings and is a common feature exhibited by plants that prefer or tolerate NH_4_^+^–N [10,39].

Different N sources can affect plant growth and biomass allocation. Our study showed that the application of NH_4_^+^–N or urea increased the plant height, root biomass and shoot biomass, which indicated that N availability can alter biomass accumulation and distribution between roots and shoots [40], and the preferred N sources for increasing biomass accumulation were NH_4_^+^–N and urea (Figure 1A,B). In this study, the higher N and chlorophyll (SPAD) levels in leaves increased the net photosynthetic rate (Figure 4A,C) and generated more carbohydrates, ultimately increasing the biomass of the above- and underground parts. In addition, our findings are also consistent with the results of Hessini et al. [13] and Khalsa et al. [7]. It was reported that NO_3_^−^–N did not contribute to the increase in biomass, which may be the photosynthetic rates were lower in the NO_3_^−^–fed plants due to the increased costs of NO_3_^−^ reduction. The roots of NO_3_^−^–fed plants exhibited decreased antioxidant enzyme activities and increased ROS and MDA levels, which may be one of the reasons why plants showed suppressed root growth [10].

Many researchers ascribe the negative effects of NH_4_^+^–N application on plants to a decrease in the pH in the root zone below five [41]. In our study, acidification and alkalization of substrates were observed in plants supplied with the NH_4_^+^-N (or urea) and NO_3_^−^–N, respectively (Table 2) [5]. When NH_4_^+^–N was supplied, the pH of the rhizosphere was the lowest at 4.45, while the EC and the content of alkaline hydrolyzed nitrogen in the root environment reached the highest values, 1.83 mS/cm and 571.2 mg/kg, respectively (Table 2). As the blackberries grew well with NH_4_^+^-N or urea, we have reason to assume that blackberries prefer an acidic environment. The reduced pH of the root zone was due to the excessive ion exchange between NH_4_^+^ and H^+^, and then H^+^ was released from root cells [41]. In addition, nitrification also leads to the release of H^+^ [42]. Electrical conductivity (EC) is an indicator of water-soluble salts, which are mineral nutrients in soils that can be quickly utilized by plants [43]. We found that NH_4_^+^ provided more abundant mineral nutrients in the cultivation substrate. Alkali-hydrolysable nitrogen (AHN) is a form of available N, and NH_4_^+^–fed plants exhibited increased available N, which may be one of the reasons that plants with the NH_4_^+^–N treatment had higher biomass, photosynthetic rate and SPAD value. In addition, inorganic N had little effect on SOC and SOM, while organic N significantly increased their content, which is the same as conventional results.

ROS (including O_2_^·−^ and H_2_O_2_) and membrane lipid peroxides (MDAs) are metabolites produced by plants during growth, and excessive amounts can trigger membrane lipid peroxidation and damage cell membranes [44]. During evolution, plants formed an antioxidant system to resist the damage caused by ROS and MDAs, which mainly includes antioxidant enzymes (SOD, POD and CAT) and nonenzymatic antioxidants such as AsA and GSH. We found that with NO_3_^−^–fed plants were more likely to induce the formation of ROS and MDA than NH_4_^+^ (or urea) –fed plants (Figure 3), leading to oxidative stress, an imbalance of oxidation and antioxidants [45] and the inhibition of root cell division and elongation. This is consistent with the findings in roots of *Spartina alterniflora* [46]. Likewise, antioxidant enzyme systems may be closely related to the N preference of plants. Plants prefer certain types of N, showing good adaptation abilities. Long-term natural selection results in plants with higher SOD, POD and CAT activities maintaining free radicals at a normal level [47]. In this study, blackberry roots exhibited higher antioxidant enzyme activities with the NH_4_^+^–N and urea treatments than with the NO_3_^−^–N treatment, which suggests that plants have a strong ability to scavenge free radicals. In addition, the activities of SOD, POD and CAT in blackberry roots were the lowest with the CK treatment, which may be due to the destruction of the antioxidant system and the inhibition of enzyme synthesis in roots under long-term nitrogen deficiency. Interestingly, when NO_3_^−^ was used as the sole N source, the levels of AsA and GSH increased significantly, which may be because the roots produced a large amount of free radicals with the NO_3_^−^–N treatment, which stimulated antioxidant action, leading to the synthesis of large amounts of antioxidants to decrease the ROS content. In addition, the soluble protein (SP) content also showed a similar trend (Figure 3I). SP is considered an important osmotic regulator that maintains the balance of osmotic potential inside and outside cells [48]. Compared with other N sources, a high accumulation of SP with the NO_3_^−^–N treatment was reported to improve the water retention capacity of cells and protect biofilms from NO_3_^−^ toxicity [49]. NH_4_^+^ significantly increased antioxidant enzyme activities and reduced ROS and MDA production in roots compared with the NO_3_^−^–N treatment. The specific mechanisms are unclear [50] but may be beneficial for enhancing antioxidant defense systems to protect plants from oxidative damage [46].

Photosynthesis and photosynthetic products such as sucrose and glucose are energy sources used to ensure that plants complete specific life activities. Photosynthesis is closely related to N availability, as N is an important component in the synthesis of chlorophyll and enzymes [51]. N availability was positively correlated with photosynthetic activity and sugar metabolism (Figure 6A), which is consistent with the responses to the NH_4_^+^–N treatment in this experiment. Our photosynthetic data showed that NH_4_^+^–fed plants had the highest net photosynthetic rate (Pn), transpiration rate (Tr) and stomatal conductance (Gs) (Figure 4C,D). This may be related to our observation of the leaf microstructure. NH_4_^+^–N can increase the number and size of stomata (Figure 2S,T), which provides the possibility of accelerating the exchange of CO_2_ and water between the cell and environment, and promote photosynthesis. In the CK group, N deficiency stress changed the leaf surface configuration, resulting in an increase in the UECs, GHs and stomatal closure (Figure 2Q,R,T), thereby reducing water evaporation, inhibiting photosynthesis and slowing metabolic activity, which is a self-protective defense mechanism in plants [52]. In addition, some researchers believe that NH_4_^+^ promotes photosynthesis because the cost of light energy to assimilate NH_4_^+^ is lower than that of NO_3_^−^ [9]. In studies on *Phaseolus vulgaris* L. [53] and sugar beets [54], the CO_2_ assimilation rate of NH_4_^+^–fed plants was higher than that of NO_3_^−^–fed plants. Our research showed that more Mg and Mn accumulated in leaves with the NH_4_^+^–N treatment (Table 2), which was beneficial to the synthesis of chlorophyll and improved the photosynthetic ability of plants. In plants, Ca mainly moves from the xylem to leaves by transpiration, and the Tr was higher in NH_4_^+^–fed plants, with higher transpiration rates leading to increased transport of calcium ions (Table 2). Notably, our experiments showed that NH_4_^+^ had a negative effect on leaf water use efficiency (LWUE), which may be due to the osmotic regulation of leaves by NH_4_^+^, which inhibited the absorption of K^+^ by leaves and ultimately reduced the water uptake rate [36]. In addition, researchers reported that in kidney beans, NH_4_^+^ significantly affected the expression of aquaporins [9]. NH_4_^+^–fed plants accumulated more sugars in roots and leaves, including glucose and sucrose (Figure 5A–D), which may be related to the higher photosynthetic rate in the NH_4_^+^–fed plants [44]. Building the C skeleton and obtaining energy (ATP) requires the breakdown of sugars for various life activities, so accumulating more sugars means the plant can better adapt to the NH_4_^+^–N environment [15].

It is well known that the long-term single application of a certain fertilizer can easily cause soil salinization. Our study showed that NH_4_^+^–N could effectively reduce the Na content in leaves (Table 1) and slow the transport of Na^+^ from the root system to the leaves, contributing to salt tolerance [55]. Ottow et al. [56] used X-ray energy spectrum analysis and found that *Populus euphratica* leaves can protect the cytoplasm from excessive Na^+^ entry after sodium ion levels reach a critical threshold. Therefore, we speculate that NH_4_^+^–fed blackberry plants may dilute a fixed amount of salt and prevent excess Na^+^ from entering the root cells.

NH_4_^+^ assimilation requires the formation of a large amount of 2-oxoglutarate from glucose, so it has been reported that tolerance to NH_4_^+^ is related to carbohydrates in roots [15]. This experiment showed that the glucose concentration in blackberry roots supplied with NH_4_^+^ was higher than that in roots supplied with NO_3_^−^, as were the sucrose and fructose concentrations (Figure 5A–C). Similar results were found in tea tree [57]. With the NH_4_^+^–N treatment, the photosynthetic rates of plants increased, as did the biomass, so the plants had more leaves (a higher-capacity source) to synthesize sugars and finally input sugars to the roots [57]. Some researchers believe that CTK is the most significant hormone affecting plant N transport, especially N regulation related to side branch formation and development [58]. Our study found that among the three N treatments, the CTK content in leaves was the highest with the NH_4_^+^–N treatment and the lowest in roots (Figure 5D), which indicated that NH_4_^+^ could promote the transport of CTK from roots to leaves, thereby promoting plant growth and development of side shoots and increasing the biomass of the aerial part (Figure 1B). At the same time, CTK has a positive effect on the distribution and transport of NH_4_^+^ and improves the N absorption and utilization rate of plants, which may explain why NH_4_^+^–fed blackberry plants can utilize N more efficiently [59]. Meier et al. [60] found that NH_4_^+^ stimulated the accumulation of IAA transported from shoots to root vascular systems and promoted the establishment of a highly branched root system. Interestingly, our study found that IAA accumulated most in the leaves with the NH_4_^+^–N treatment, possibly contributing to the growth of the aboveground parts. Moreover, this is a highly complex regulatory process in which IAA and CTK jointly participate, and the response mechanism remains to be further studied.

Amino acid metabolism in blackberry is not only the central link of N metabolism but also plays an important role in the regulation of cell osmosis and ROS detoxification [14,61]. In general, the content of free amino acids in N-supplied blackberry plants was significantly higher than that in N-deficient blackberry plants, especially with the supply of NH_4_^+^, and there were certain differences in the accumulation of amino acids in roots and leaves. As a sole N source, NH_4_^+^ may accumulate in large amounts in leaves, which is usually toxic to plants [14]. The massive accumulation of proline and soluble sugars in blackberry leaves may be a strategy for detoxifying excess NH_4_^+^ [62]. Soluble sugars can maintain leaf turgor, and they collaborate with amino acids to maintain cell membrane structure and function under abiotic stresses [63]. In roots, the levels of most of the amino acids, such as proline, aspartic acid and threonine [64,65], with the NO_3_^−^–N treatment were significantly higher than those with the other treatments (*p* < 0.05). This suggests that the levels of osmotic regulators may increase in stressed roots to protect the plants from osmotic stress; the root system was indeed in an unfavorable environment, as evidenced by the minimal biomass and accumulated MDA with the NO_3_^−^–N treatment. Arginine (Arg), an amino acid used for N transport and storage, also promotes root development [66]. Our study showed that NH_4_^+^ could increase the Arg content in roots and leaves, similar to the results of Carr et al. [25]. Notably, the content of Arg in roots was more than 10 times that in leaves. This showed that the high accumulation of Arg in roots did not prevent the timely assimilation and transport of NH_4_^+^ and promoted N transport from the roots to the aerial parts; excess N was stored as Arg, which is a manifestation of blackberry tolerance to NH_4_^+^ [25].

**Figure 7:**
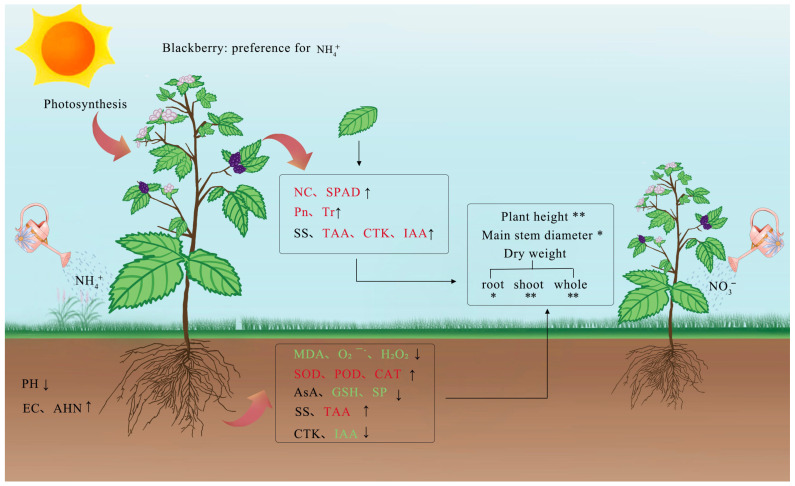
Physiological characteristics of whole blackberry plants with different N forms affected blackberry growth and the associated correlations: a possible physiological regulatory mechanism. The red font indicates that an indicator has a significant positive correlation with blackberry growth and the green font indicates a negative correlation. The upward arrow indicates that the value of this indicator increases with the NH_4_^+^–N treatment relative to the NO_3_^−^–N treatment, that is, NH_4_^+^/NO_3_^−^ > 1; the downward arrow indicates the opposite. * represents *p* < 0.05, and ** represents *p* < 0.01.

To understand the correlation between the various indicators and determine which indicators had a large impact on plant growth, we studied the correlation between the physiological response to different N forms and the growth of blackberry plants by correlation analysis and PCA. This investigation showed that the levels of MDA and ROS (O_2_^·−^, H_2_O_2_) were significantly negatively correlated with antioxidant enzyme activities, and the photosynthetic parameters were significantly positively correlated with the antioxidant enzyme activities and IAA and amino acid levels (Figure 6A). Two principal components were extracted from 21 physiological indicators, of which PC1 was significantly associated with blackberry growth (Figure 6A, Table 4 and Table 5). To elucidate the physiological regulatory mechanism of blackberry growth more intuitively under different N conditions, we drew a mechanistic diagram, and the interconnections and regulatory relationships are shown in Figure 7.

## 4. Materials and Methods

### 4.1. Plant Material, Growth Conditions, and Treatments

One-year-old cutting seedlings of blackberry cultivar ‘Ningzhi 4’ (Kiowa × Hull) of the same size with no insect pests were used. The seedlings were supplied by Nanjing Baima National Agricultural Science and Technology Park. The experiment was performed in a greenhouse at the Institute of Botany, Jiangsu Province and Chinese Academy of Sciences (32°3′10.01′′ N, 118°49′58.22′′ E) with an average temperature of 29/16 °C (day/night) and a relative humidity between 55% and 80%.

Notably, the rapid growth period of blackberry plants is mainly from May to August, and the colonization period is mainly from March to April. After August, the number of leaves gradually declines, and the aboveground parts gradually die, while the underground parts can live for many years. Our experiments were conducted to study vegetative growth throughout the growth period. In March 2021, the selected cutting seedlings were transplanted into plastic pots (top diameter 30 cm, bottom diameter 24 cm, height 33 cm) with pure coconut peat (pH 5.0, C/N 80) as a culture substrate. After colonization, 40 d of adaptive precultivation was carried out with NPK full nutrient solution (Shandong Jining Jinshan Biological Engineering Co., Ltd., Jining, China). Subsequently, a 20-d fertilizer control treatment (water only) was carried out. In May, the seedlings were divided into four groups, namely, the nitrogen deficiency group (CK), ammonium nitrogen group (NH_4_^+^-N), nitrate nitrogen group (NO_3_^−^-N) and organic nitrogen group (urea). During the test, the seedlings received different N forms, and the total N supply concentration was 15 mM [19]. Each treatment was set up in nine pots as repeats, completely randomly arranged. The CK group received a modified Hoagland nutrient solution without N, and the other elements remained unchanged. The nutrient solution (excluding N) contained 588 mg/L CaCl_2_·2H_2_O, 136 mg/L KH_2_PO_4_, 372.8 mg/L KCl, 493 mg/L MgSO_4_·7H_2_O, 6.2 mg/L H_3_BO_3_, 16.9 mg/L MnSO_4_·H_2_O, 36.7 mg/L EDTA·FeNa, 0.83 mg/L KI, 8.6 mg/L ZnSO_4_·7H_2_O, 0.25 mg/L Na_2_MoO_4_·2H_2_O, 0.025 mg/L CuSO_4_·5H_2_O and 0.025 mg/L CoCl_2_·6H_2_O. The N source in the NH_4_^+^-N group was replaced by (NH_4_)_2_SO_4,_ and that in the NO_3_^−^-N group was replaced by Ca(NO_3_)_2_·4H_2_O and NaNO_3_. We used CH_4_N_2_O (urea) as an organic N source. In order to prevent the nitration of ammonium and urea, the nitration inhibitor dicyandiamine (DCD) was added to the matrix [9]. During seedling cultivation, the nutrient solution (400 mL per pot) was added twice per week (previously, we have conducted a preliminary experiment, and this amount of N fertilizer was found to be sufficient and suitable for the growth of blackberry plants). The experiment ended in August, and we harvested whole plants.

### 4.2. Assessment of Plant Growth, Biomass and Morphology

The plant height and main stem diameter were measured using tape and a digital Vernier caliper. The shoots and roots were separately wrapped in paper bags and placed in a drying oven at 80 °C until a constant weight was reached for dry weight determination.

On the 60th day after the experimental treatment, the roots and top fully expanded leaves were cut into small pieces (1.5 × 2 mm) and fixed immediately in 4% (*v*/*v*) glutaraldehyde with 0.2 mol/L phosphate buffer (pH 7.8). The samples were further dehydrated in a series of tert-butyl alcohols and lyophilized. Finally, the samples were gold-sputtered and observed using a QUANTA 200 environmental scanning electron microscope (FEI-Co., Ltd., Hillsboro, OR, USA). Five fields of view were randomly selected to count the number of UECs, GHs, stomata and open stomata. In addition, qualitative and quantitative analyses of elements were carried out on the cross section of the leaf blade by using an X-ray energy spectrometer (FEI-Co., Ltd., USA) equipped with a scanning electron microscope.

### 4.3. Determination of the Physical and Chemical Properties of the Cultivation Substrate

#### 4.3.1. Measurement of pH and EC

The pH and EC of the cultivation substrate were measured by the potentiometric method and electrode method, respectively. Approximately 1 g of dried substrate (passed through a 100-mesh sieve) was weighed and placed in a 50 mL tube. Approximately 10 mL of water was added to dissolve the substrate, followed by centrifugation at 8000 rpm for 10 min at 4 °C. The supernatant was collected and tested, the pH was measured using a pH meter and the EC was measured using a conductivity meter.

#### 4.3.2. Determination of Organic Matter

The potassium dichromate volumetric method is widely applied to estimate the SOM content. The dried substrate (0.01 g) was poured into a glass test tube, and 5 mL 0.8 mol/L 1/6 K_2_CrO_7_ standard solution and 5 mL concentrated sulfuric acid were added. The solution was heated to 180 °C in an oil bath and boiled for 5 min. Then, it was titrated with 0.2 mol/L FeSO_4_, and the solution changed from orange–yellow to blue–green and then to brown–red as the end point of the titration.

(1) $$SOC(\%)=\frac{\frac{0.8\times 5.0}{V_{0}}\times (V_{0}-V)\times 0.003\times 1.1}{m_{1}\times K_{2}}\times 100$$

(2) SOM(%)=SOC(%)×1.724

where 0.8 (mol/L) is the concentration of 1/6 K_2_CrO_7_ standard solution and 5.0 (mL) is its volume; $V_{0}$ is the volume of FeSO_4_ solution for blank calibration, mL; V is the volume of FeSO_4_ solution used to titrate the sample, mL; 0.003 is the molar mass in kg of 1/4 carbon atoms; 1.1 is the oxidation correction coefficient; 1.724 is the coefficient used to convert organic carbon into organic matter; $m_{1}$ is the mass of the dried substrate sample, g; $K_{2}$ is the conversion factor to convert moist substrate to air-dried substrate; and SOC represents substrate organic carbon.

#### 4.3.3. Determination of Alkali-Hydrolysable N

Alkali-hydrolysable N was determined by the alkaline hydrolysis diffusion method [11]. The dried substrate (0.5 g) was evenly placed into the outer chamber of the diffusion dish. Approximately 2 mL of 2% boric acid solution and 1 drop of N indicator were added to the inner chamber of the diffusion dish. The outer edge of the diffusion dish was coated with Arabic glue and covered with a ground glass lid. The glass lid was pushed aside, 10 mL of 1.2 mol/L NaOH was quickly added and the ground glass lid was closed. Rubber bands were used to hold the ground glass lid in place and the diffusion dish was placed in a 40 °C incubator for 24 h. Finally, the solution was titrated with 0.01 mol/L standard hydrochloric acid solution.

(3) $$AHN(\mathrm{mg}/\mathrm{kg})=\frac{\left(V-V_{0}\right)\times C\times 14\times 1000}{W}$$

where $V_{0}$ is the volume of hydrochloric acid consumed in the blank experiment, mL; V is the volume of hydrochloric acid consumed by the sample, mL; 14 represents the grams of 1 mol N; 1000 represents the grams of N converted into a 1 kg sample; C is the standard concentration of hydrochloric acid; and W is the mass of dried substrate sample, g.

### 4.4. Measurement of Root Physiology and Antioxidant Indicators

The malondialdehyde (MDA) content in blackberry roots was determined by the thiobarbituric acid (TBA) method [67]. The superoxide anion radical (O_2_^·−^) generation rate was determined according to the hydroxylamine oxidation reaction method [68]. The hydrogen peroxide (H_2_O_2_) content was determined using a kit (A064-1-1, Nanjing Jiancheng Bioengineering Institute, Nanjing, China). The SOD activity was determined by the nitro blue tetrazolium (NBT) method [69]. POD activity was determined by catalytic hydrogen peroxide reactions [23]. The amount of enzyme that catalyzed 1 microgram of substrate per minute per gram of tissue was defined as a unit of POD activity (U) at 37 °C. The CAT activity was determined by the ammonium molybdate method [70]. One unit of CAT activity (U) was defined as the amount of 1 μM H_2_O_2_ decomposed per second per gram of tissue. The ascorbic acid (AsA) content was determined according to the method of Asami et al. [71], and the reduced glutathione (GSH) content was determined according to the method of Anderson [72]. The SP content was determined using Coomassie brilliant blue staining [73].

### 4.5. Analysis of Photosynthetic Parameters

The relative chlorophyll content (SPAD) and N content (usually the upper fully expanded leaves) were monitored every three days with a plant nutrition measurement instrument (TYS-4N, Zhejiang TOP Cloud-agri Technology Co., Ltd., Hangzhou, Zhejiang, China). Approximately 30 leaves in each treatment were selected for observation for 60 days.

In mid-July (when the weather was sunny), the photosynthetic parameters were measured using an LI-6800 photosynthesis instrument (Beijing Ecotek Technology Co., Ltd., Beijing, China) on mature leaves of robust branches from 8:00 to 11:00. Approximately 5 leaves were selected per plant, and 4 seedlings were selected per treatment. In addition, the atmospheric temperature (AT) and atmospheric CO_2_ concentration (Ca) were measured every hour on that day. The light intensity during the measurement was approximately 1500 μmol/(m^2^·s).

### 4.6. Determination of the Sugar and Endogenous Hormone Contents in Roots and Leaves

The sucrose, fructose, glucose and soluble sugar levels were estimated using a testing kit (Nanjing Jiancheng Bioengineering Company, Nanjing, China). CTK and IAA levels were determined by enzyme-linked immunosorbent assays (ELISAs) [74]. The fresh tissue (0.2 g) was ground in 80% methanol at 4 °C for 12 h and then centrifuged at 10,000 rpm for 15 min. This process was repeated twice, and the supernatants were dried with a N_2_ blow and rotary evaporator. The freeze-dried remaining substances were dissolved in phosphate buffer (containing 0.1% (v v^−1^) Tween-20 and 0.4% (m v^−1^) NaCl; pH 7.5) and then analyzed by ELISA.

### 4.7. Assessment of Free Amino Acid Contents in Roots and Leaves

The free amino acid levels were determined according to the method of Zhou et al. [75]. Approximately 0.2 g of dry sample (passed through a 0.25 mm sieve) was added to a conical flask and brewed with 10 mL of boiled water. The mixture was heated and shaken in a water bath at 95 °C and extracted for 20 min. Then, vacuum filtration was performed while the mixture was still hot to obtain a filtrate. The filtrate was vacuum freeze-dried (FD5 freeze dryer, SIM, USA). After drying, dilution buffer was added to dissolve the filtrate, which was passed through a 0.22 µm filter membrane, and free amino acids were detected using an S433D automatic amino acid analyzer (Sykam, Eresing, Germany). The analysis conditions were as follows: chromatographic column, LCA K06/Na; mobile phase A, 0.012% citric acid-sodium citrate buffer, pH 3.45; mobile phase B, 0.02% citric acid-sodium citrate, pH 10.85; 58–74 °C, gradient temperature control; flow rate elution pump, 0.45 mL/min + derivatization pump 0.25 mL/min; pressure, 3–4 MPa; UV detection wavelengths, 570 and 440 nm.

### 4.8. Statistical Analysis

All measurements are presented as the mean ± SD of 3 replicates (photosynthetic parameters *n* = 20). The data were input and analyzed by Excel 2010 software and IBM SPSS Statistics 25.0 software (IBM Corp., Armonk, NY, USA). The charts were produced by Excel 2010, Origin 2021 (Origin Lab Inc., San Francisco, CA, USA) and Adobe Photoshop 2020 (Photoshop Software, San Diego, CA, USA). The growth parameters, photosynthetic index, antioxidant system, soluble proteins, sugars, amino acids, hormones and the physical and chemical properties of substrates were analyzed by the general linear model, which is a fixed effect model. Normal distribution and homogeneity test of variance were conducted on samples before ANOVA processing, which all met the above conditions. One-way ANOVA was performed for significance testing (*p* < 0.05). Duncan’s test was used to compare the means of all paired measurement values. Pearson correlation coefficient was used for correlation analysis. Principal component analysis (PCA) analysis was performed with Origin 2021 (Origin Lab Inc., USA). Kaiser-Meyer-Olkin (KMO) test was conducted on sample variables, and KMO > 0.7, so PCA could be conducted.

## 5. Conclusions

N fertilizer is an important factor affecting the growth and development of the blackberry plant. With the continuous expansion of blackberry cultivation areas in the world, farmers tend to apply excessive N fertilizer to increase yields. However, the unreasonable selection of N fertilizer not only makes blackberry unable to absorb and utilize N normally, but also causes toxicity to plants and pollutes the environment. Based on our findings, the blackberry plant seems to be NH_4_^+^–tolerant and NH_4_^+^–preferring plants at the seedling stage, and its adaptability to NH_4_^+^ is better than that to NO_3_^−^. Urea is another suitable choice, which has a similar effect as NH_4_^+^–N. NH_4_^+^ can significantly promote the growth of blackberry and enhance antioxidant enzyme activities and photosynthesis. In addition, NH_4_^+^-fed plants were conducive to the accumulation of osmoprotectants such as soluble sugars and amino acids, which could be a strategy to detoxify excess NH_4_^+^. In contrast, NO_3_^−^ inhibited root growth and the plants accumulated more reactive oxygen species and MDA. From the point of view of growers, blackberry seedlings should consider increasing NH_4_^+^ in a fertilization program and using less NO_3_^−^. Our study could provide a practical basis for blackberry fertilization management for sustainable agriculture in the future.

## Figures and Tables

**Table 1:** Quantitative analysis results obtained by EDS with different N treatments.

	CK		NH_4_^+^–N		NO_3_^−^–N		Urea	
Element	Weight%	Atomic%	Weight%	Atomic%	Weight%	Atomic%	Weight%	Atomic%
C	37.21	45.92	35.26	44.64	39	49.57	38.98	50.21
O	51.33	47.55	51.17	48.64	45.72	43.63	43.76	42.31
Na	8.35	5.38	5.82	3.85	6.82	4.53	6.28	4.22
Mg	0.65	0.4	1.27	0.79	−	−	0.53	0.34
K	−	−	−	−	−	−	0.59	0.23
Ca	0.98	0.36	3.45	1.31	0.89	0.34	1.74	0.67
Mn	0.9	0.24	1.32	0.37	-	-	-	-
Fe	0.44	0.12	−	−	2.65	0.73	1.63	0.45
Cu	0.14	0.03	−	−	1.57	0.41	4.05	0.99
Zn	−	−	1.71	0.4	2.1	0.5	2.44	0.58
Mo	−	−	−	−	1.25	0.29	−	−
Total	100	100	100	100	100	100	100	100

Processing option: All elements analyzed (normalized). Number of iterations = 3. The symbol “−” indicates that the instrument did not detect the element.

**Table 2:** Physical and chemical properties of the cultivation substrate with different N forms.

Treatment	pH	EC (mS/cm)	SOM (%)	SOC (%)	AHN (mg/kg)
CK	5.37 ± 0.01 b	0.96 ± 0.01 c	74.02 ± 0.70 b	42.93 ± 0.41 b	402.27 ± 14.09 d
NH₄^+^–N	4.45 ± 0.01 d	1.83 ± 0.02 a	72.26 ± 1.39 b	41.91 ± 0.80 b	571.20 ± 12.83 a
NO₃^−^–N	6.25 ± 0.01 a	0.97 ± 0.017 c	72.02 ± 1.20 b	41.78 ± 0.69 b	481.60 ± 2.80 c
Urea	5.00 ± 0.02 c	1.25 ± 0.07 b	80.72 ± 0.68 a	46.82 ± 0.39 a	522.67 ± 9.00 b

EC, electrical conductivity; SOM, substrate organic matter; SOC, organic carbon; AHN, alkali-hydrolysable nitrogen. The data indicated are the means ± SDs (*n* = 3). Different values in the same column indicate significant differences between different treatments (*p* < 0.05).

**Table 3:** Concentrations of free amino acids in blackberry roots and leaves with different N treatments.

	Root/mg·(100 g)^−1^ DW				Leaf/mg·(100 g)^−1^ DW			
Amino Acid	CK	NH₄^+^–N	NO₃^−^–N	Urea	CK	NH₄^+^–N	NO₃^−^–N	Urea
Asp	1.30 ± 0.11 c	1.82 ± 0.02 b	2.33 ± 0.04 a	1.40 ± 0.09 c	1.22 ± 0.18 d	6.81 ± 0.55 a	4.53 ± 0.40 b	3.86 ± 0.17 c
Thr	0.91 ± 0.08 c	1.23 ± 0.05 b	1.52 ± 0.01 a	0.84 ± 0.11 c	1.00 ± 0.15 c	3.24 ± 0.38 a	2.39 ± 0.32 b	3.36 ± 0.12 a
Ser	5.08 ± 0.45 c	11.46 ± 0.65 a	11.28 ± 0.10 a	7.77 ± 0.78 b	1.48 ± 0.15 d	19.80 ± 1.99 a	10.20 ± 0.97 c	15.57 ± 0.39 b
Glu	1.56 ± 0.16 c	1.92 ± 0.06 b	3.60 ± 0.18 a	1.82 ± 0.19 bc	1.99 ± 0.08 b	2.29 ± 0.25 ab	2.75 ± 0.24 a	2.31 ± 0.12 ab
Gly	0.94 ± 0.18 a	0.51 ± 0.06 b	0.66 ± 0.13 b	0.46 ± 0.08 b	0.30 ± 0.06 c	0.92 ± 0.05 a	0.39 ± 0.03 c	0.68 ± 0.03 b
Ala	2.32 ± 0.19 c	2.93 ± 0.08 b	3.47 ± 0.01 a	2.10 ± 0.23 c	4.04 ± 0.42 d	16.30 ± 1.31 a	10.78 ± 1.19 c	13.63 ± 0.42 b
Cys	0.08 ± 0.01 b	0.16 ± 0.01 a	0.17 ± 0.01 a	0.15 ± 0.02 a	0.08 ± 0.01 c	0.66 ± 0.03 a	0.40 ± 0.03 b	0.64 ± 0.04 a
Val	0.76 ± 0.07 d	1.48 ± 0.08 b	1.80 ± 0.02 a	1.01 ± 0.08 c	1.34 ± 0.18 c	6.10 ± 0.77 a	5.05 ± 0.26 b	6.02 ± 0.18 a
Met	0.15 ± 0.03 c	0.38 ± 0.01 a	0.33 ± 0.02 b	0.16 ± 0.01 c	0.11 ± 0.01 c	0.88 ± 0.12 a	0.57 ± 0.06 b	0.71 ± 0.01 b
Ile	0.45 ± 0.03 c	0.93 ± 0.04 a	1.05 ± 0.00 a	0.57 ± 0.08 b	1.11 ± 0.14	5.12 ± 0.46	3.7 ± 0.64	5.03 ± 0.13
Leu	0.33 ± 0.04 c	0.60 ± 0.05 b	1.00 ± 0.05 a	0.48 ± 0.11 bc	1.24 ± 0.16 c	5.40 ± 0.50 a	4.34 ± 0.33 b	5.59 ± 1.18 a
Tyr	0.74 ± 0.09 a	0.60 ± 0.05 b	0.78 ± 0.02 a	0.50 ± 0.03 b	1.39 ± 0.05 c	4.96 ± 0.32 a	4.09 ± 0.44 b	3.97 ± 0.09 b
Phe	1.77 ± 0.01 b	1.28 ± 0.18 d	2.09 ± 0.01 a	1.53 ± 0.11 c	3.37 ± 0.18 c	10.21 ± 0.82 b	12.49 ± 1.06 a	11.99 ± 0.35 ab
His	1.72 ± 0.02 c	3.59 ± 0.08 a	2.36 ± 0.06 b	1.73 ± 0.13 c	1.45 ± 0.15 c	5.89 ± 0.42 a	4.34 ± 0.47 b	4.44 ± 0.36 b
Lys	0.38 ± 0.01 d	0.88 ± 0.02 a	0.73 ± 0.02 b	0.51 ± 0.05 c	0.66 ± 0.13 c	2.63 ± 0.27 ab	2.53 ± 0.20 b	3.07 ± 0.10 a
Arg	3.61 ± 0.02 d	34.61 ± 1.26 a	8.1 ± 0.35 c	11.08 ± 1.34 b	0.39 ± 0.04 d	3.39 ± 0.37 a	1.74 ± 0.19 c	2.64 ± 0.12 b
Pro	1.08 ± 0.13 c	1.64 ± 0.10 b	2.2 ± 0.15 a	1.02 ± 0.15 c	1.31 ± 0.17 d	13.82 ± 0.59 a	6.28 ± 0.50 c	8.50 ± 0.23 b
∑EAA	6.47 ± 0.28 b	10.37 ± 0.16 a	10.86 ± 0.12 a	6.84 ± 0.46 b	10.27 ± 1.02 c	39.26 ± 2.21 a	35.41 ± 2.65 b	40.21 ± 1.23 a
∑NEAA	16.71 ± 1.14 d	55.84 ± 0.74 a	32.58 ± 0.67 b	26.29 ± 2.78 c	12.20 ± 1.12 d	68.94 ± 4.79 a	41.15 ± 3.74 c	51.78 ± 1.26 b
∑TAA	23.17 ± 1.47 d	66.01 ± 0.78 a	43.45 ± 0.59 b	33.13 ± 3.08 c	22.46 ± 1.97 d	108.20 ± 7.02 a	76.56 ± 4.09 c	91.99 ± 2.27 b

The data indicated are the means ± SDs (*n* = 3). All data are expressed as mg/100 g DW. Different letters in the same row indicate significant differences between treatments (*p* < 0.05). Asp, aspartic acid; Thr, threonine; Ser, serine; Glu, glutamic acid; Gly, glycine; Ala, alanine; Cys, cysteine; Val, valine; Met, methionine; Ile, isoleucine; Leu, leucine; Tyr, tyrosine; Phe, phenylalanine; His, histidine; Lys, lysine; Arg, arginine; Pro, proline. Essential amino acids (∑EAA): Thr + Val + Met + Ile + Leu + Phe + His + Lys; nonessential amino acids (∑NEAA): Asp + Ser + Glu + Pro + Gly + Ala + Cys + Tyr + Arg. Total amino acids (∑TAA): ∑EAA + ∑NEAA.

**Table 4:** Eigenvalues of each principal component.

Trait	Component	
	1	2
MDA	−0.88 **	−0.05
O_2_^·−^	−0.77 **	−0.14
H_2_O_2_	−0.74 **	0.60
SOD	0.89 **	−0.42
POD	0.90 **	0.18
CAT	0.95 **	−0.23
AsA	−0.43	0.64 **
GSH	−0.63 **	0.11
SP	−0.94 **	−0.06
SPAD	0.91 **	0.39
NC	0.95 **	0.31
Pn	0.98 **	−0.01
Tr	0.95 **	−0.22
RSS	−0.48	−0.87 **
LSS	−0.69	−0.72 **
RCTK	−0.40	0.66 **
LCTK	0.73 **	0.19
RIAA	−0.88 **	0.30
LIAA	0.88 **	−0.15
RTAA	0.81 **	−0.04
LTAA	0.96 **	0.25
Total	14.01	3.28
% of variance	66.72	15.64
Cumulative %	66.72	82.36

** represents eigenvalues that are significant, i.e., >0.60.

**Table 5:** Correlation coefficients between the principal components and growth indexes.

Index	PC1	PC2	Plant Height	Main Stem Diameter	Root Dry Weight	Shoot Dry Weight	Whole Dry Weight
PC1	1	0	0.888 **	0.588 *	0.556 *	0.863 **	0.837 **
PC2	0	1	0.353	0.057	0.770 **	0.476	0.517

* represents *p* < 0.05 and ** represents *p* < 0.01.

## Data Availability

The data generated for this study are available on request to the corresponding author.
